# Supplementary material for: The Q61H mutation decouples KRAS from upstream regulation and renders cancer cells resistant to SHP2 inhibitors
Source: Nat Commun. 2021 Nov 1;12:6274. doi: 10.1038/s41467-021-26526-y (PMC8560773; doi:10.1038/s41467-021-26526-y)
Supplement: Supplementary file 1 — Supplementary Information [file 41467_2021_26526_MOESM1_ESM.pdf]

The Q61H mutation decouples KRAS from upstream regulation and renders cancer cells resistant to SHP2 inhibitors

Gebregiworgis et al.

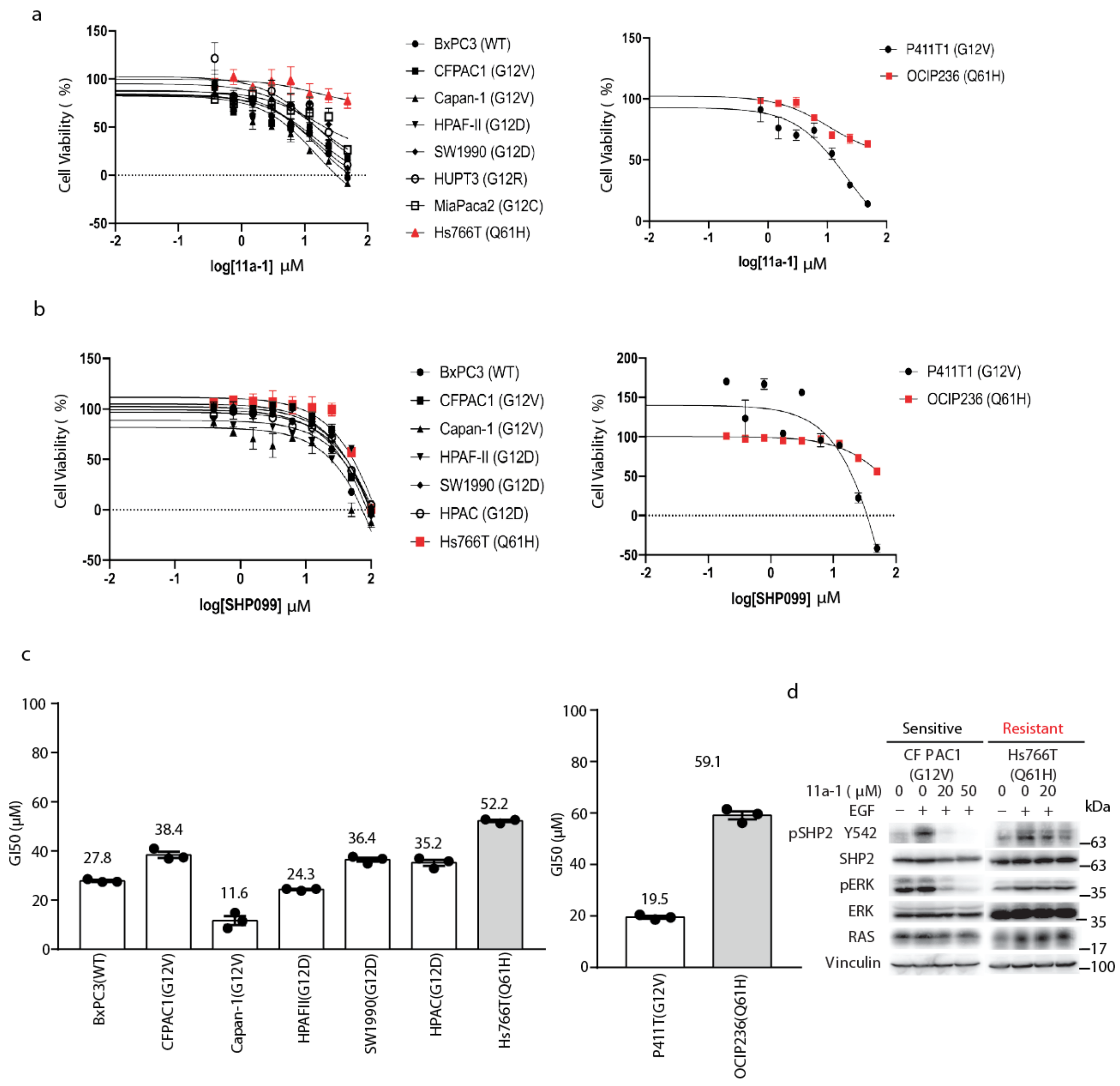

Supplementary Fig. 1: Pancreatic cancer cells harboring KRAS Q61H are resistant to SHP2 inhibitors. (**a**) PDAC cell lines (left) or PDX cells (right) were plated in 96-well plates in triplicate, treated with increasing concentrations of 11a-1 for 48 h and then cell viability was determined using alamarBlue. The dots represent mean of three independent experiments for each cell line at each inhibitor concentration. The error bars indicate s.e.m of three independent experiments. GI50 values derived from these plots are shown in Figure 1b. (**b**) PDAC cell lines (right) or PDX cells (left) were plated in 96-well plates in triplicate, treated with increasing concentrations of SHP099 for 48 h and then cell viability was determined using alamarBlue. Individual points are shown as in panel a. (**a, b**) (**c**) GI50 values from the data in panel b determined using GraphPad Prism 7.0. Data represent mean ± s.e.m. of three independent experiments. KRAS mutation

status of each cell type is indicated. (**d**) Pancreatic cancer cells (CF PAC1 or Hs766T) were serum starved and pretreated with (+) or without (-) the indicated concentrations of 11a-1 for 5 h and then treated with 10ng/ml of EGF for 5 min. Equal amounts of lysates were resolved on SDS-PAGE and immunoblotted with antibodies recognizing phosphorylated ERK and SHP2, as well as the indicated antibodies. The immunoblot data are representative of at least three independent experiments.

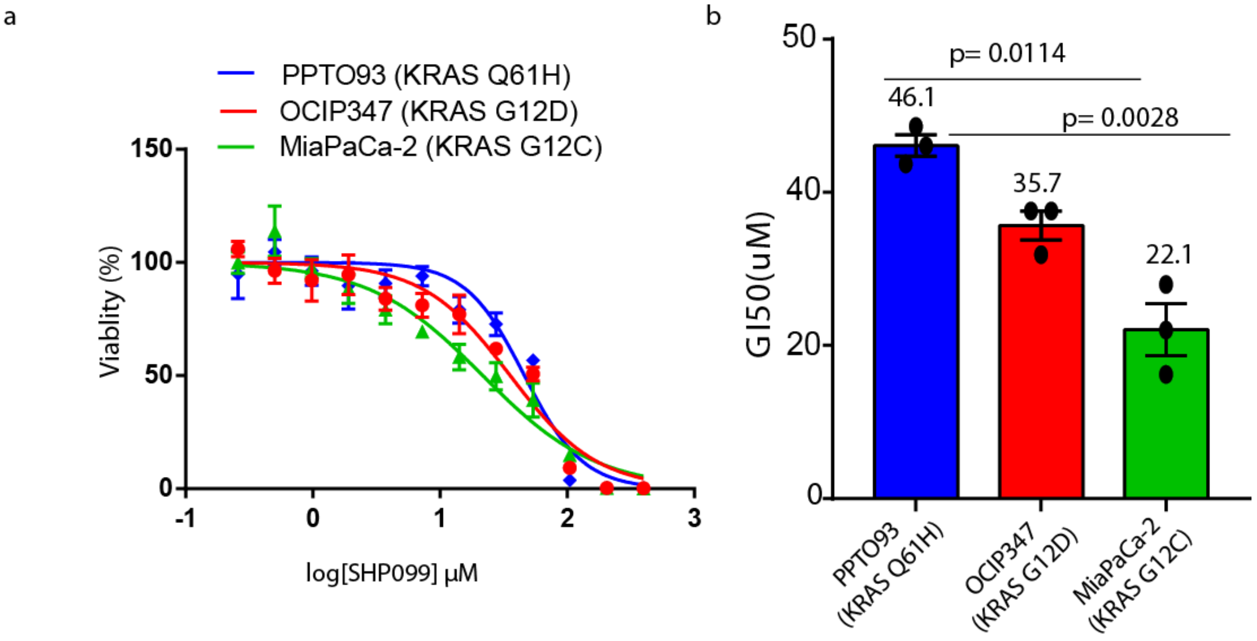

Supplementary Fig. 2: A patient derived pancreatic organoid with KRASQ61H mutation is less sensitive to SHP099 treatment. (a) PDAC cell line or PD organoids cells were plated in 96-well plates in triplicate, treated with increasing concentrations of SHP099 for 96 h and then cell viability was determined using Titer-Glo. Individual points are shown as the mean of three experiments with error bars representing s.d. **(b)** GI50 values from the data in panel a determined using GraphPad Prism 7.0. Data represent mean ± s.e.m of three independent experiments. The p-values were obtained using two-tailed Student's t-test.

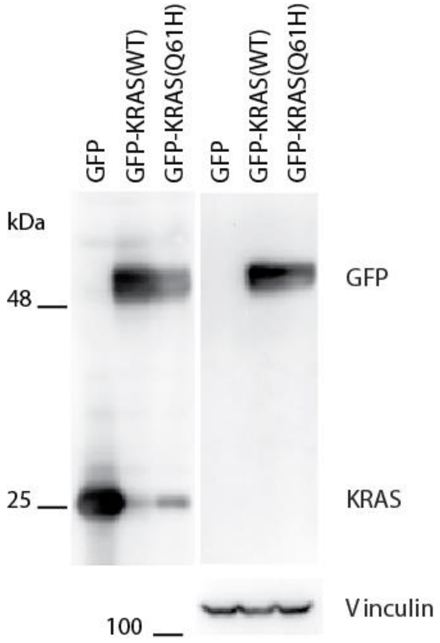

Supplementary Fig. 3: Confirmation of stable overexpression of GFP-KRAS W.T and Q61H in HEK293 cells for evaluation of resistance to SHP2 inhibitor treatment. Equal amounts of lysates of isogenic HEK293 cells stably over-expressing GFP-KRAS WT, GFP-KRAS Q61H or GFP alone were resolved on SDS-PAGE and immunoblotted with anti-GFP and anti-KRAS antibodies to verify expression. The blots are representative of three independent experiments.

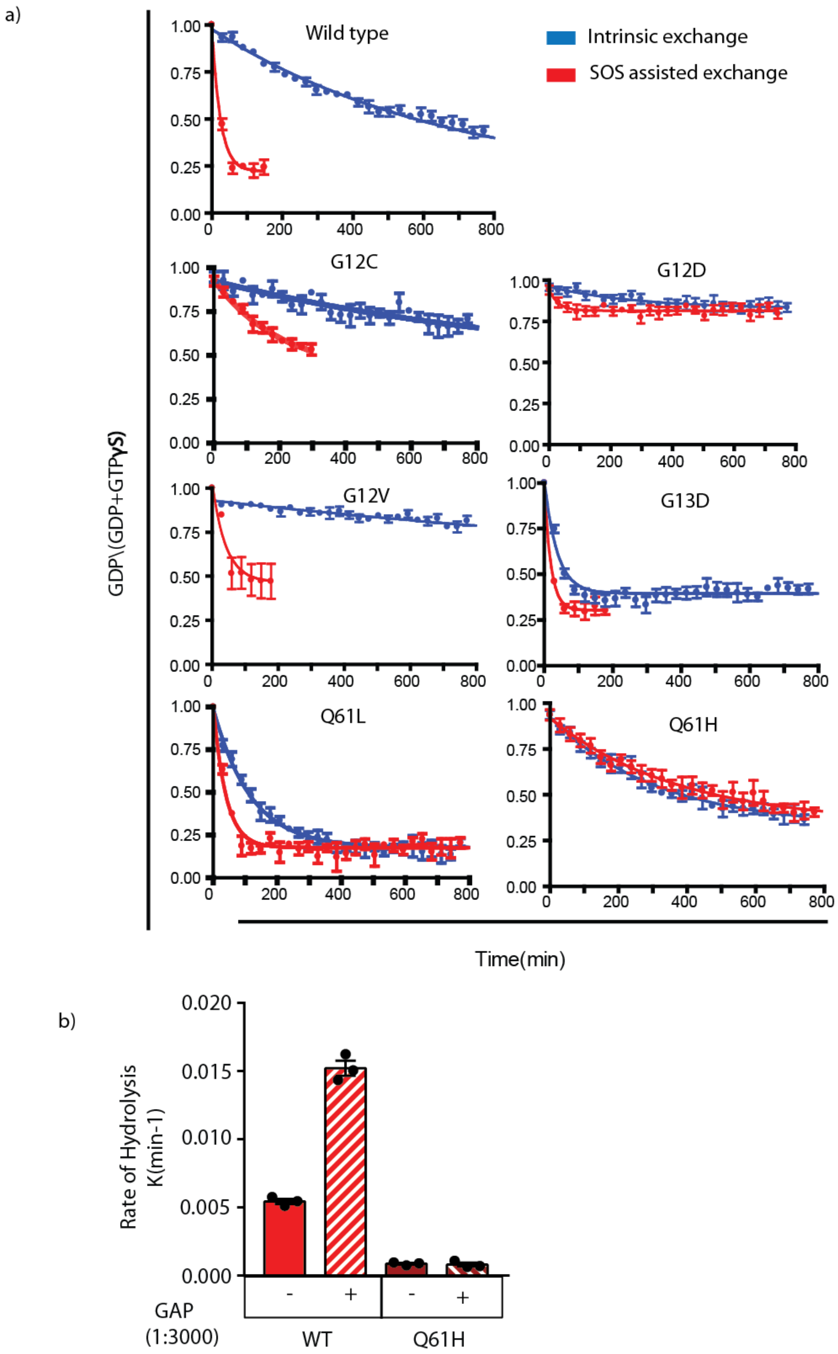

Supplementary Fig. 4: KRAS Q61H is resistant to GTPase cycle regulation by SOS and RASA1. (a) Intrinsic (blue) and SOS^{cat}-assisted (red) nucleotide exchange curves for wild-type KRAS and six common mutants (G12V, G12C, G12D, G13D, Q61L and Q61H) obtained by real-time NMR. A 10-fold excess of GTP analog (guanosine 5'-O-[gamma-thio] triphosphate) was added to 250 µM ^{15}N-labeled KRAS-GDP and sequential ^{1}H-^{15}N HSQC NMR spectra were collected as exchange proceeded. The dots represent the fraction of KRAS that is GDP loaded based on the intensities of GDP- and GTP-specific KRAS peaks from three residues, the error

bars represent standard deviation of the intensities of the three peaks. The data were fitted to one-phase exponential decay functions using GraphPad Prism 7. For SOS-assisted exchange assays, SOS^{cat} was added at a 1:600 molar ratio to KRAS. The derived exchange rates are plotted in Figure 2 b and d. **(b)** Histogram of the intrinsic and RASA1 GAP-assisted GTP hydrolysis rates of wild-type, and KRAS mutants (Q61H and Q61L), which were obtained from real-time NMR assays presented in Figure 2g. Fully GTP-loaded ^{15}N KRAS samples (250 µM, WT or Q61H) were placed in an NMR tube and GTP hydrolysis was monitored in the absence (-, intrinsic rate) or presence (+) of RASA1 GAP domain (1:3000 molar ratio). intensity measurements of three residues. The error bars represent s.e.m of rates obtained from peak intensity measurement of three residues.

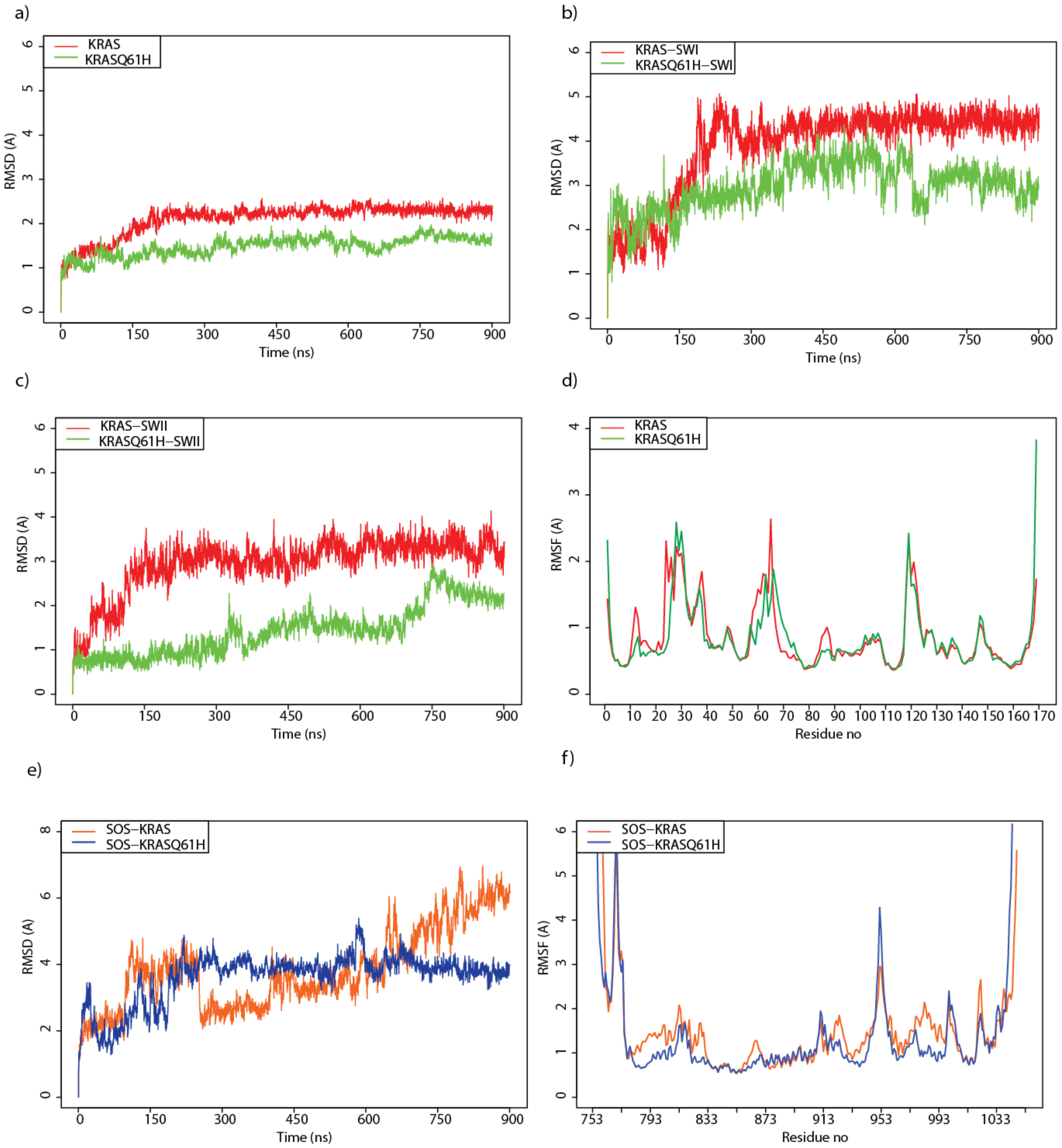

Supplementary Fig. 5: **The Q61H mutation alters dynamics of KRAS and its interaction with cc. (a-c)** Root Mean Square Deviation (RMSD) of alpha carbons of wild-type KRAS (red) and KRAS Q61H (green) for **(a)** all residues (1 to 169), **(b)** switch I residues (27 to 37), and **(c)** switch II residues (58 to 65). **(d)** Root Mean Square Fluctuation (RMSF) of wild-type KRAS (red) and KRAS Q61H (green). **(e)** RMSD and **(f)** RMSF of SOS_{cat} bound to wild-type KRAS (orange) and KRAS Q61H (blue).

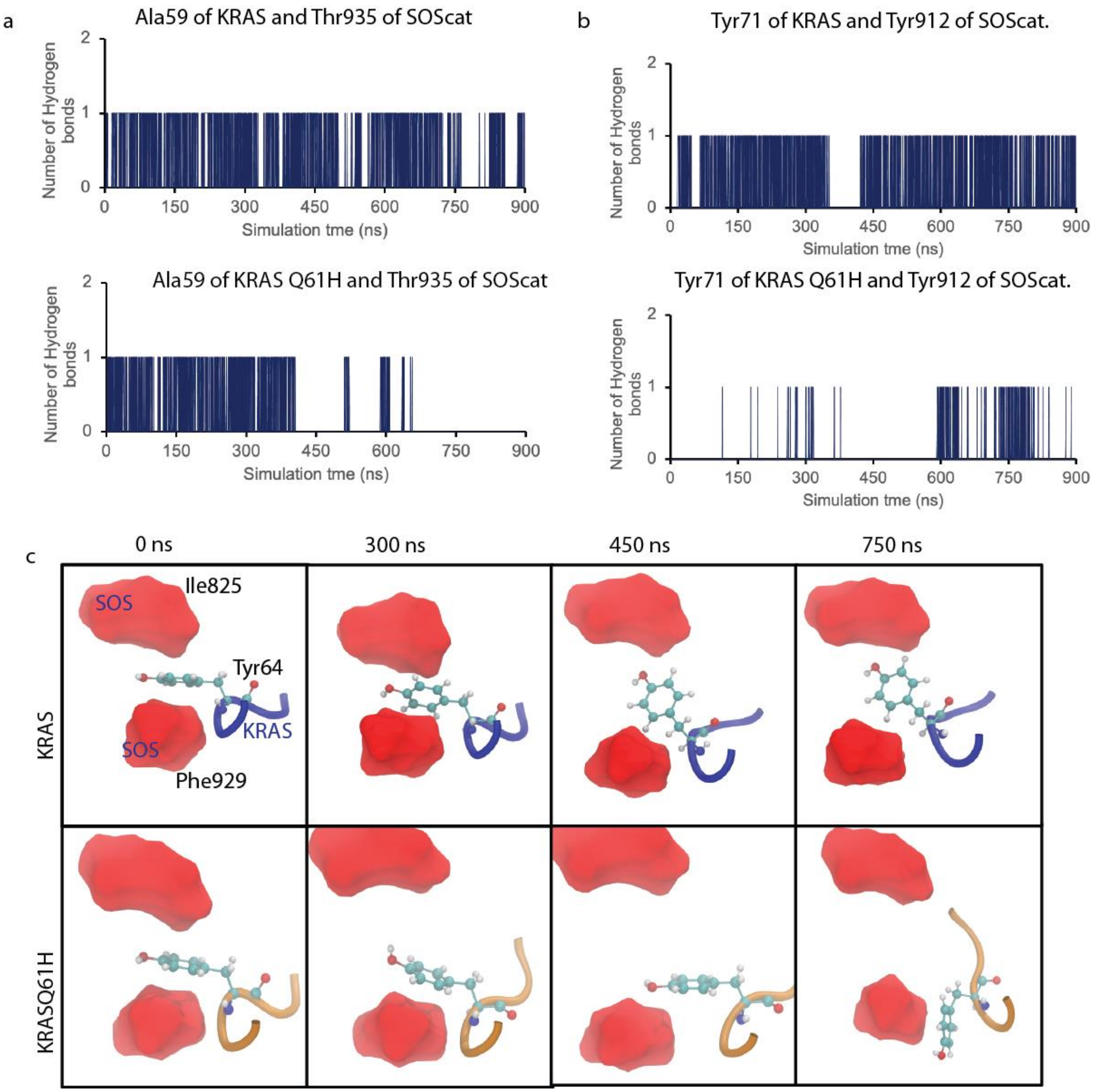

Supplementary Fig. 6: **KRAS Q61H mutation reduces the number of interactions between the switch II region of KRAS and SOS**. Time evolution representation of the number of hydrogen bonds formed between **(a)** Ala59 of KRAS and Thr935 of SOS_{cat} **(b)** Tyr71 of KRAS and Tyr912 of SOScat. Top plots represent wild-type KRAS and bottom plots are for KRAS Q61H. The data presented was obtained from frames collected at 300 ps intervals of the 900 ns simulation. Hydrogen bonds were defined using a donor to acceptor distance and an angle cutoff of 3 Ǻ and 20°, respectively. **(c)** Cartoon representation depicting the hydrophobic interaction between

Tyr64 of KRAS (top, wild type; bottom, Q61H) and Ile825 and Phe929 of SOS_{cat} at different intervals of the trajectory (0 ns, 300 ns, 450 ns, and 750 ns).

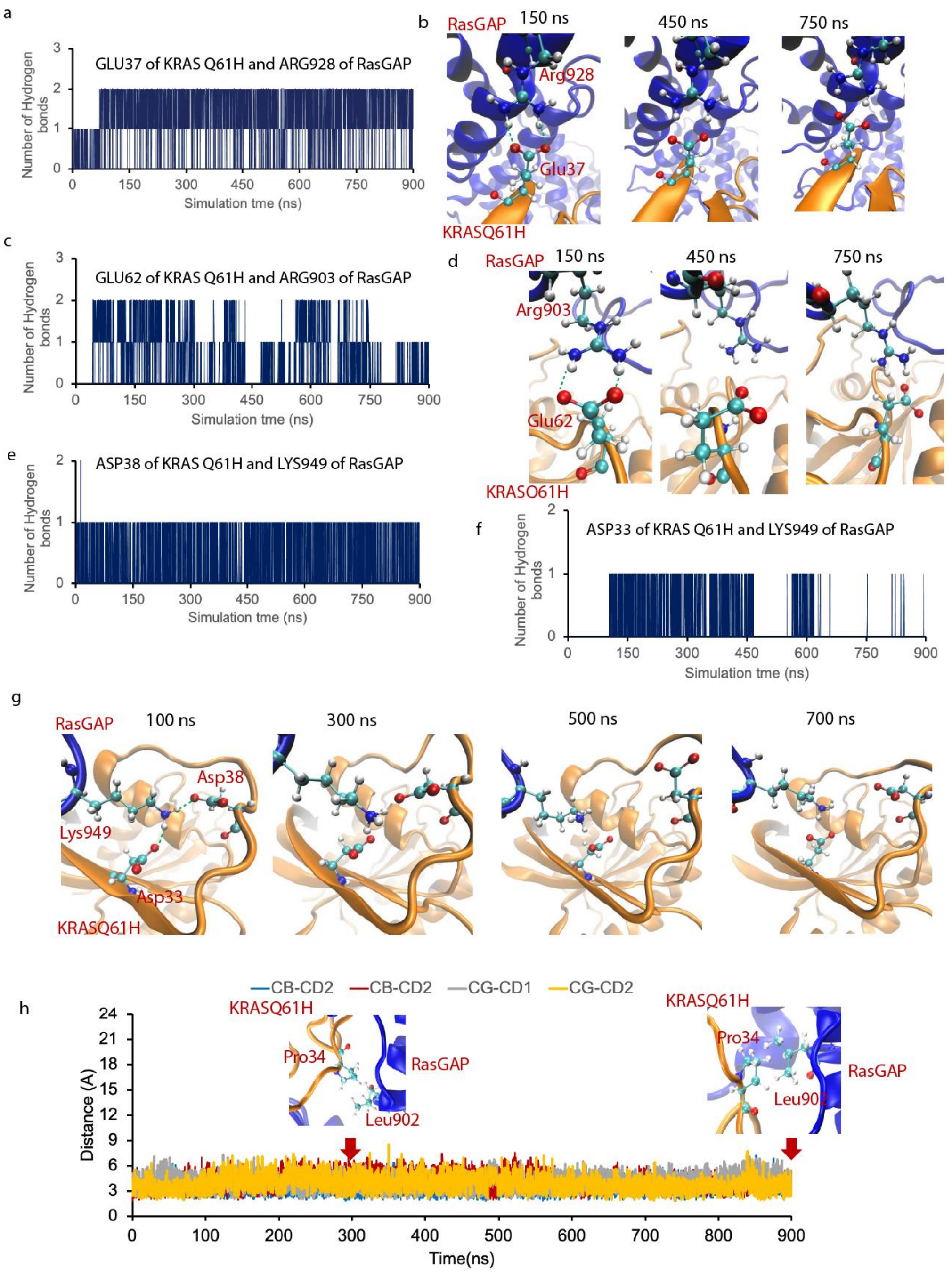

Supplementary Fig. 7: Polar interactions between KRAS Q61H and RASGAP. Time evolution representation of the number of hydrogen bonds formed between **(a)** GLU37 of KRAS Q61H and ARG928 of RasGAP (RASA1) **(b)** Representative frames that depict the orientations of GLU37 of KRAS Q61H and ARG928 of RasGAP **(c)** GLU62 of KRAS Q61H and ARG903 of RasGAP. **(d)** GLU62 of KRAS Q61H and ARG903 of RasGAP at different simulation times (150, 450, and 750 ns). Time evolution representation of number of hydrogen bonds formed between LYS949 of RasGAP and **(e)** ASP38, or **(f)** ASP33 of KRAS Q61H. (**g)** Representative frames that

depict the orientation of ASP38 and ASP33 of KRAS Q61H and LYS949 of RasGAP at different time points (100 ns, 300 ns, 500 ns, 700 ns). **(h)** Time evolution distance between beta and gamma carbons of LEU902 (RASGAP) and delta carbons of PRO34 (KRASQ61H), in the colours as indicated. The data presented was obtained from data collected at 300 ps intervals of the 900 ns simulation. Hydrogen bonds were defined using a donor to acceptor distance and an angle cutoff of 3 Ǻ and 20°, respectively. Selected hydrogen bonds are indicated with dotted green lines.

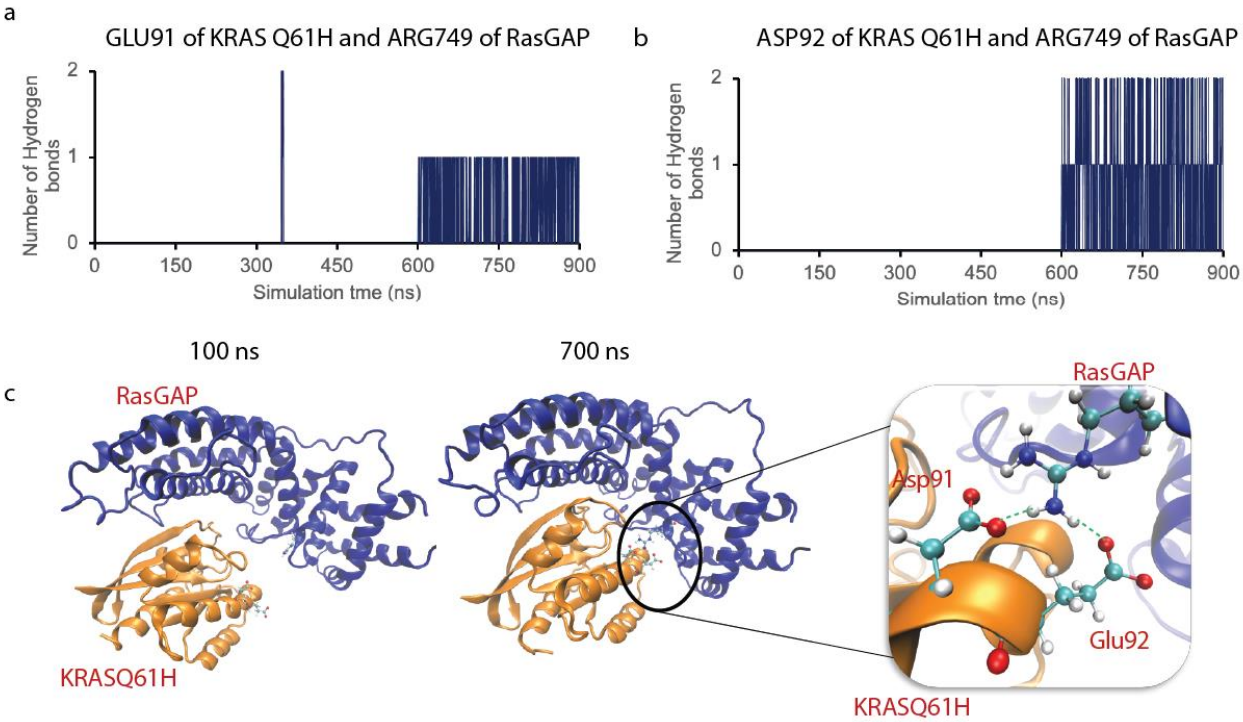

.

Supplementary Fig. 8: A polar interaction is predicted to form between KRAS Q61H and the extra domain of RasGAP. Time evolution representation of the number of hydrogen bonds formed between **(a)** GLU91 or **(b)** ASP92 of KRAS Q61H and ARG749 in the extra domain of the Ras GAP (RASA1). **(c)** Representative cartoons depicting the relative orientations of KRAS Q61H and RasGAP (snapshots at 100 and 700 ns). The three residues that establish a new interaction between the two proteins are shown as balls and sticks in the cartoons, with a zoomed view of GLU91, and ASP92 (KRAS Q61H) and ARG749 (RasGAP). Hydrogen bonds were defined using a donor to acceptor distance and an angle cutoff of 3 Ấ and 20°, respectively. Selected hydrogen bonds are indicated with dotted green lines.

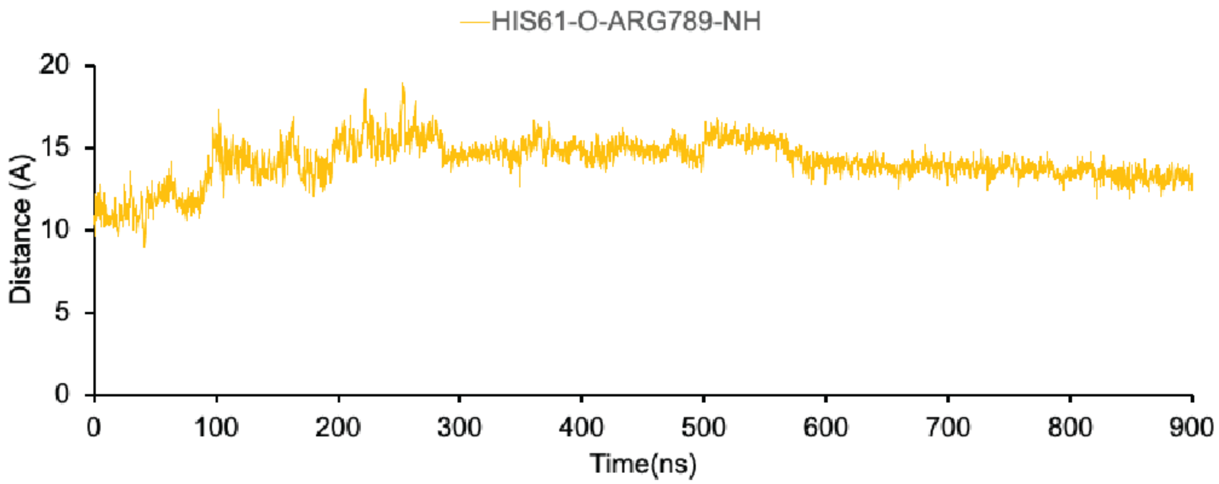

Supplementary Fig. 9: KRAS Q61H mutation alters the catalytic site. Time evolution representation of the distance between backbone carboxyl of His61 (KRAS) and backbone NH of Arg789 (RasGAP).

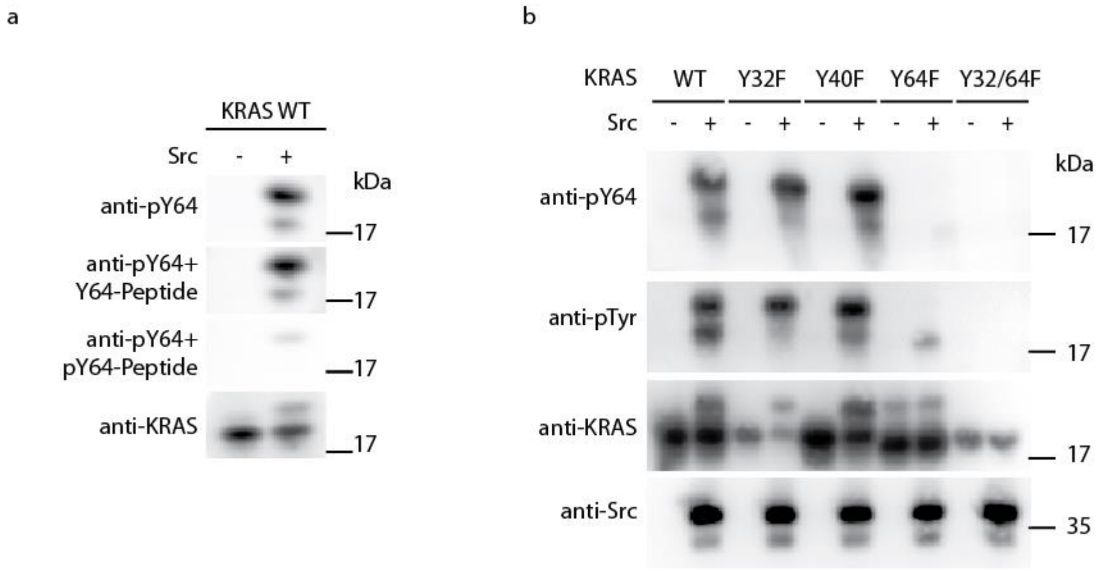

Supplementary Fig. 10: Validation of phospho-Tyr64 specific RAS antibody. (a) GTP-bound KRAS WT was phosphorylated in vitro using purified recombinant Src kinase domain, and immunoblotted with the newly developed anti-pY64 KRAS antibody or anti-KRAS. Where indicated, the pY64 antibody was incubated with 5-fold excess (by weight) of blocking peptide for 30 min at room temperature prior to immunoblotting. pY64, phospho-Tyr64-specific RAS antibody; Y64-peptide, 12-mer peptide comprising the KRAS sequence flanking pTyr64 (TAGQEEYSAMRD); pY64-peptide, tyrosyl phosphorylated- Y64 peptide. **(b)** GTP-bound KRAS WT, Y32F, Y40F, Y64F, and Y32/64F were phosphorylated in vitro using purified Src kinase domain, and immunoblotted with indicated antibodies. The blots are representative of three independent experiments.

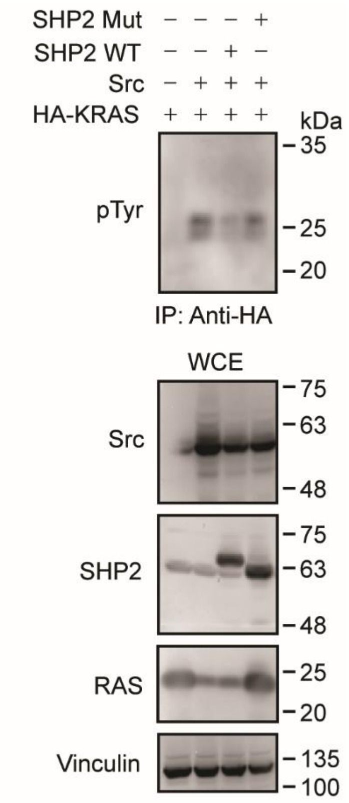

Supplementary Fig. 11: SHP2 reduces tyrosyl phosphorylation of KRAS. HEK293 cells were co-transfected with plasmids encoding wild-type SHP2 or a catalytically dead mutant (C459S) together with Src and wild-type HA-KRAS as indicated. HA-KRAS was immune precipitated from the respective cell lysates and immunoblotted with anti-phosphotyrosine antibody. The blots are representative of three independent experiments.

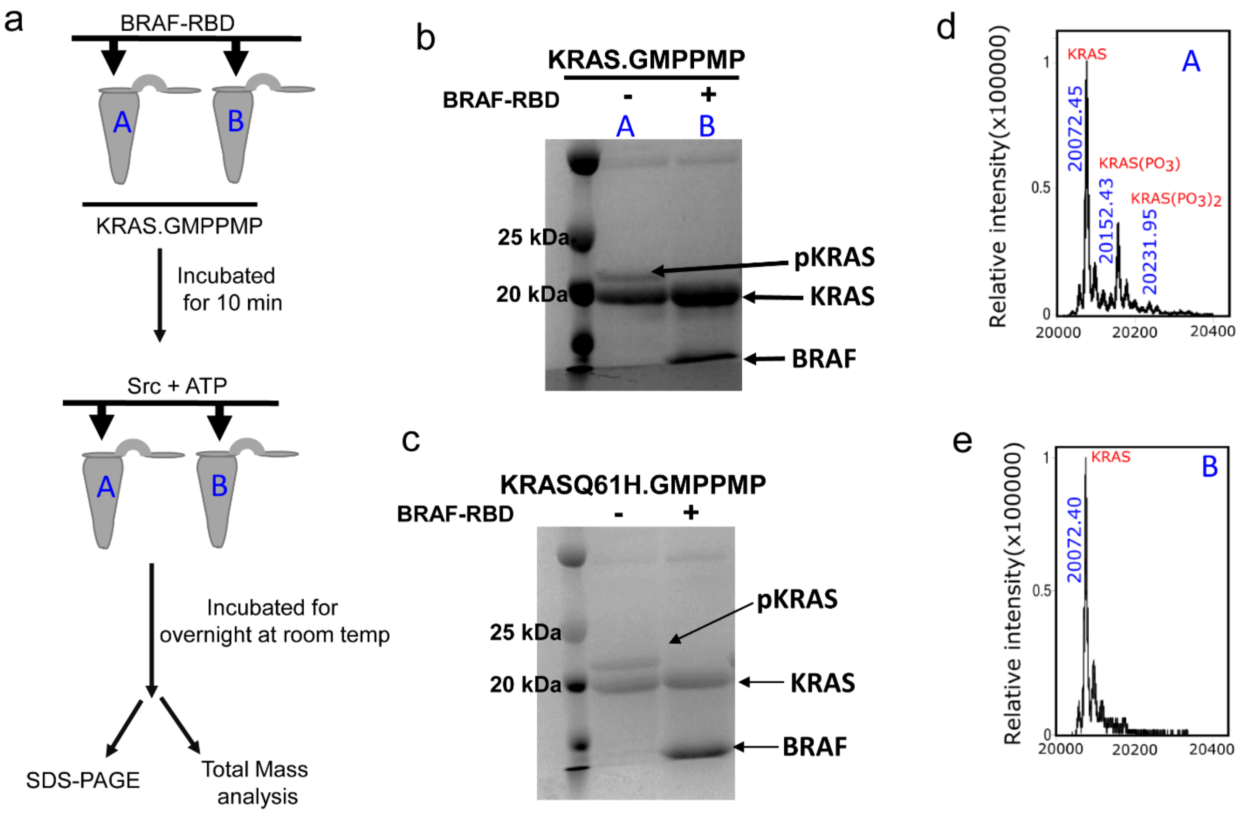

Supplementary Fig. 12: Binding of the BRAF-RBD to activated KRAS blocks phosphorylation by Src. **(a)** Cartoon depicting the in vitro phosphorylation experiment: KRAS bound to GMPPNP (a non-hydrolysable GTP analogue) was incubated with two-fold molar excess BRAF-RBD for 10 min (tube B) or alone (tube A). Then the catalytic domain of Src (1:125 molar ratio), 2mM ATP and phosphatase inhibitor cocktail were added to each tube, and they were incubated at room temperature overnight. Finally, the samples were analyzed by SDS-PAGE and mass spectrometry. **(b)** Coomassie Brilliant Blue-stained SDS-PAGE analyses for tubes A and B. The unmodified KRAS protein runs as a single band near the 20 kDa marker, whereas phosphorylated KRAS migrates more slowly and produces a distinct band. (c) SDS-PAGE analyses of GMPPMP-loaded KRAS Q61H following exposure to Src +/-BRAF-RBD as performed for wild-type KRAS. **(d,e)** Mass spectrometry analyses of samples A and B (as indicated). The parent mass for ^{15}N labeled KRAS is ~ 20,072 Da. The masses of mono- and di-phosphorylated KRAS are ~20,152 and ~20,232 Da, respectively. (b,c) the SDS-PAGE gels are representative of at least three independent experiment.

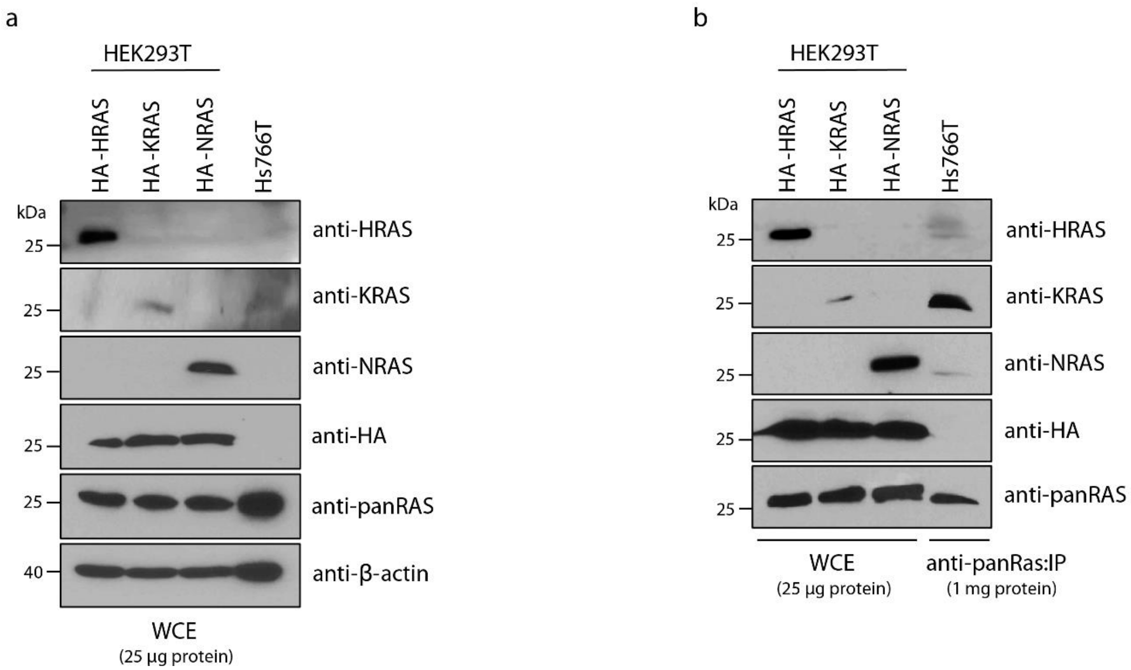

Supplementary Fig. 13: Hs766T cells predominantly express KRAS isoform. (a) HEK293 cells were transfected with HA-HRAS WT, HA-KRAS WT or HA-NRAS WT plasmids as indicated. These transfected HEK293T cells and Hs766T cells were cultured for 48 h. Equal amounts of lysates were resolved on SDS-PAGE and immunoblotted with the indicated antibodies to assess expression of RAS isoforms. WCE, whole cell extract. **(b)** Hs766T cell lysates were immunoprecipitated (IP) using anti-panRAS antibody and probed with the indicated antibodies recognizing each RAS isoform. Equal amounts of HEK293 cell lysates overexpressing HA-HRAS WT, HA-KRAS WT or HA-NRAS WT plasmids, which were used as controls for specificity and quality of the antibodies, were resolved on SDS-PAGE and blotted alongside Hs766T precipitated lysates. (**a, b**) The blots are representative of three independent experiments.

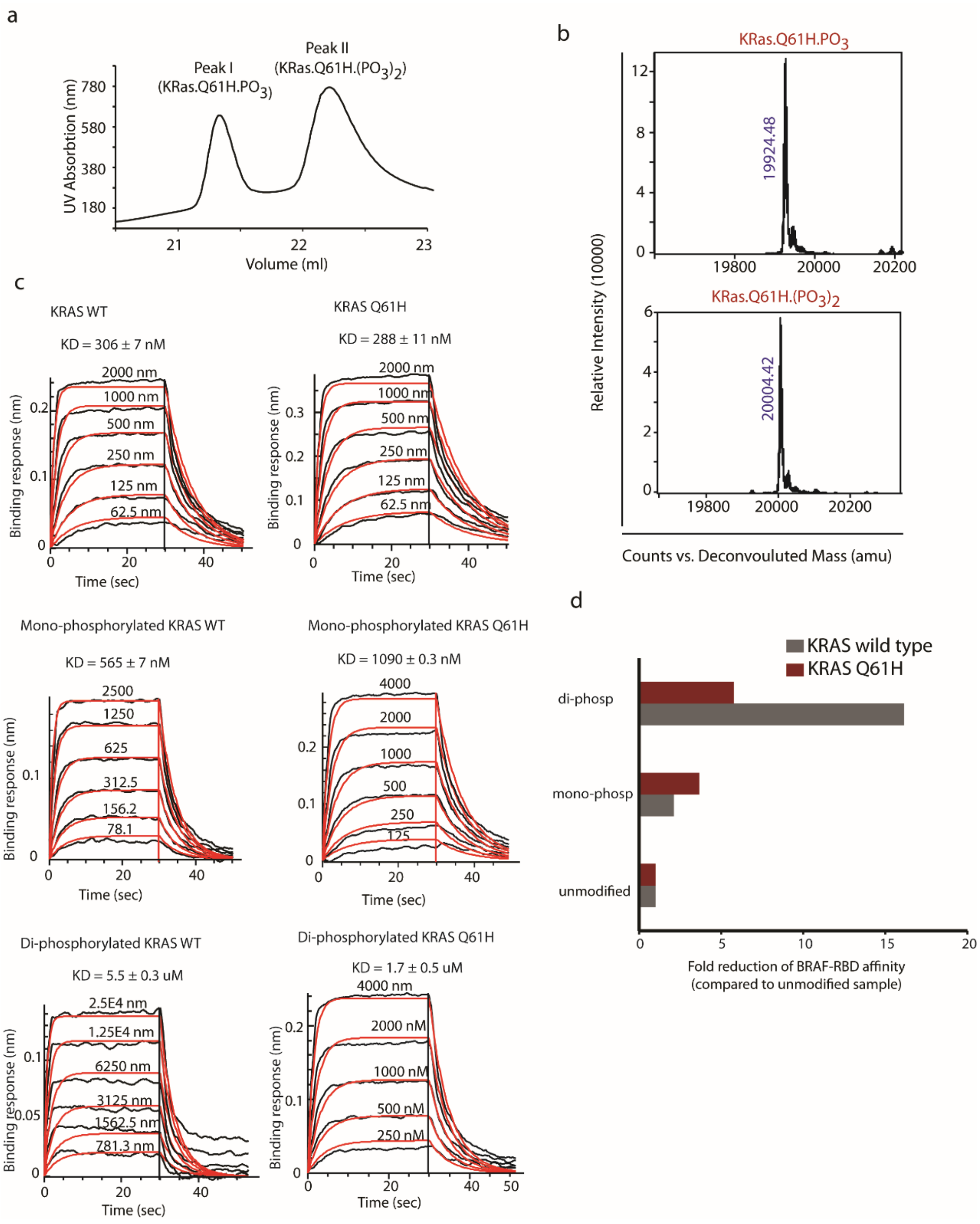

Supplementary Fig. 14: Src phosphorylation of KRAS has differential impact on wild-type versus Q61H BRaf RBD binding (a) Separation of mono- and di-phosphorylated KRAS Q61H using ion-exchange chromatography. In anion exchange, the mono-phosphorylated form elutes first followed by di-phosphorylated KRAS Q61H. A Mono Q 5/50 GL column was run with 20 mM HEPES pH 7.0, 5 mM MgCl2, and 1mM TCEP (Buffer A), and 20 mM HEPES pH 7.0, 5 mM MgCl2, 1mM TCEP, and 1M NaCl (Buffer B) using a gradient of 0 to 40% B over 80 column

volumes. A phosphatase inhibitor cocktail was added to both buffers. **(b)** Mass spectra of intact mono- and di-phosphorylated KRAS Q61H samples. The predicted mass of KRAS Q61H (residues 1-173, C118S) is 19844.36 Da. Upper panel, mono-phosphorylated fraction (+80 Da); lower panel, di-phosphorylated fraction (+160 Da). **(c)** Biolayer interferometry (Octet) analyses of binding of KRAS WT and Q61H to immobilized BRAF RBD. Unmodified, mono-phosphorylated and di-phosphorylated KRAS WT and Q61H were used at the indicated concentrations. The measured binding curves are black, and fitted curves are red. Kd values determined by kinetic analysis are shown. **(d)** Histogram comparing the fold reduction in BRAF-RBD binding affinity caused by phosphorylation (mono- and di-) of wild-type KRAS versus the Q61H mutant.

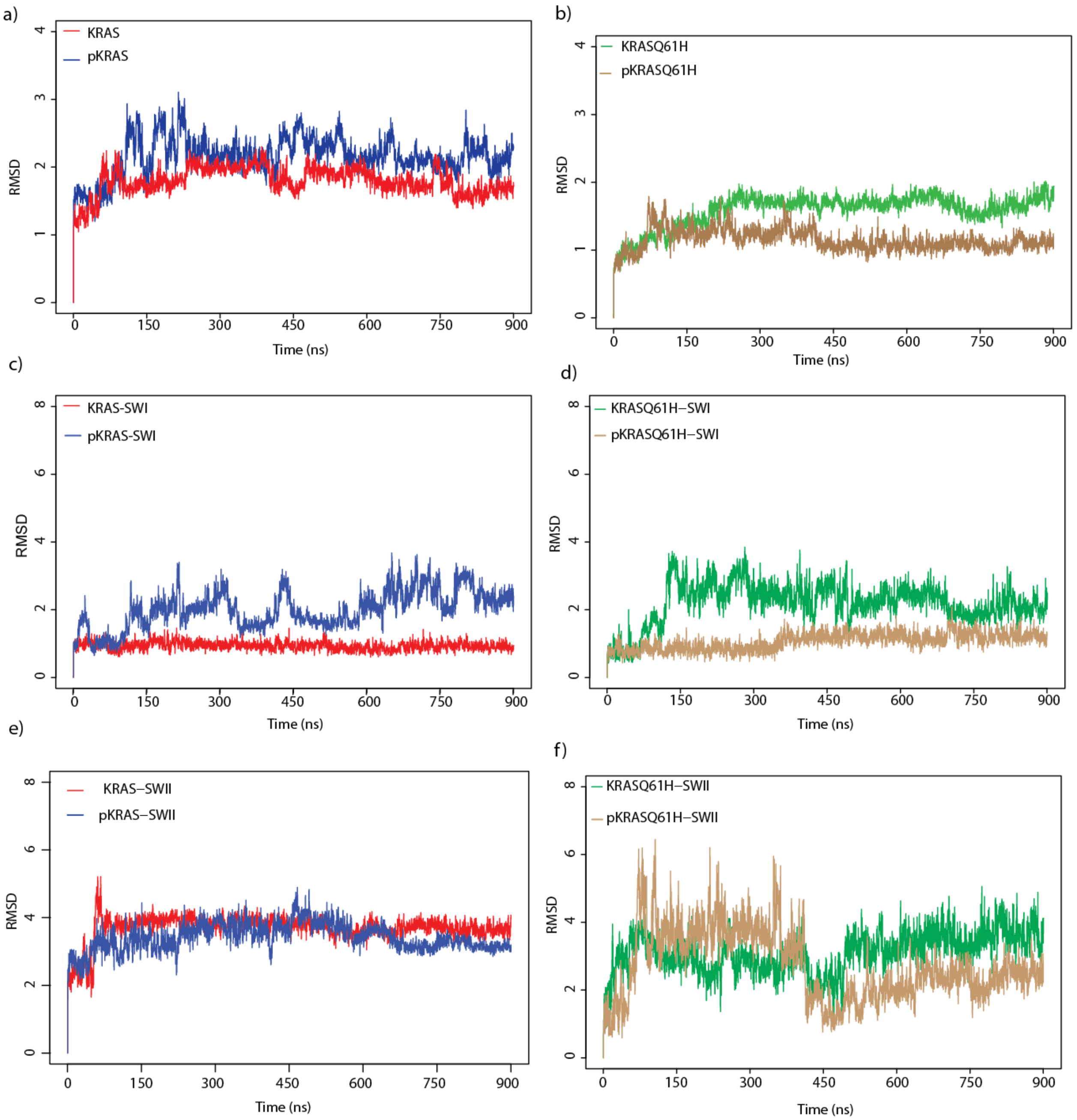

Supplementary Fig. 15: RMSD measurements of WT-KRAS and the Q61H mutant (unmodified and tyrosyl phosphorylated) in complex with RAF1. RMSD measurements of the indicated components of KRAS in the MD simulations of **(a, c, e)** wild-type KRAS, unmodified (red), or phosphoryolated at Y32 and Y64 (blue), and **(b, d, f)** KRAS Q61H mutant, unmodified (green), or phosphoryolated at Y32 and Y64 (brown). The graphs show the trajectory of RMSD for (a, b), the GTPase domain, **(c, d)** switch I, and **(e, f)** switch II. The data presented was obtained from data collected at 300 ps intervals throughout the 900 ns simulation.

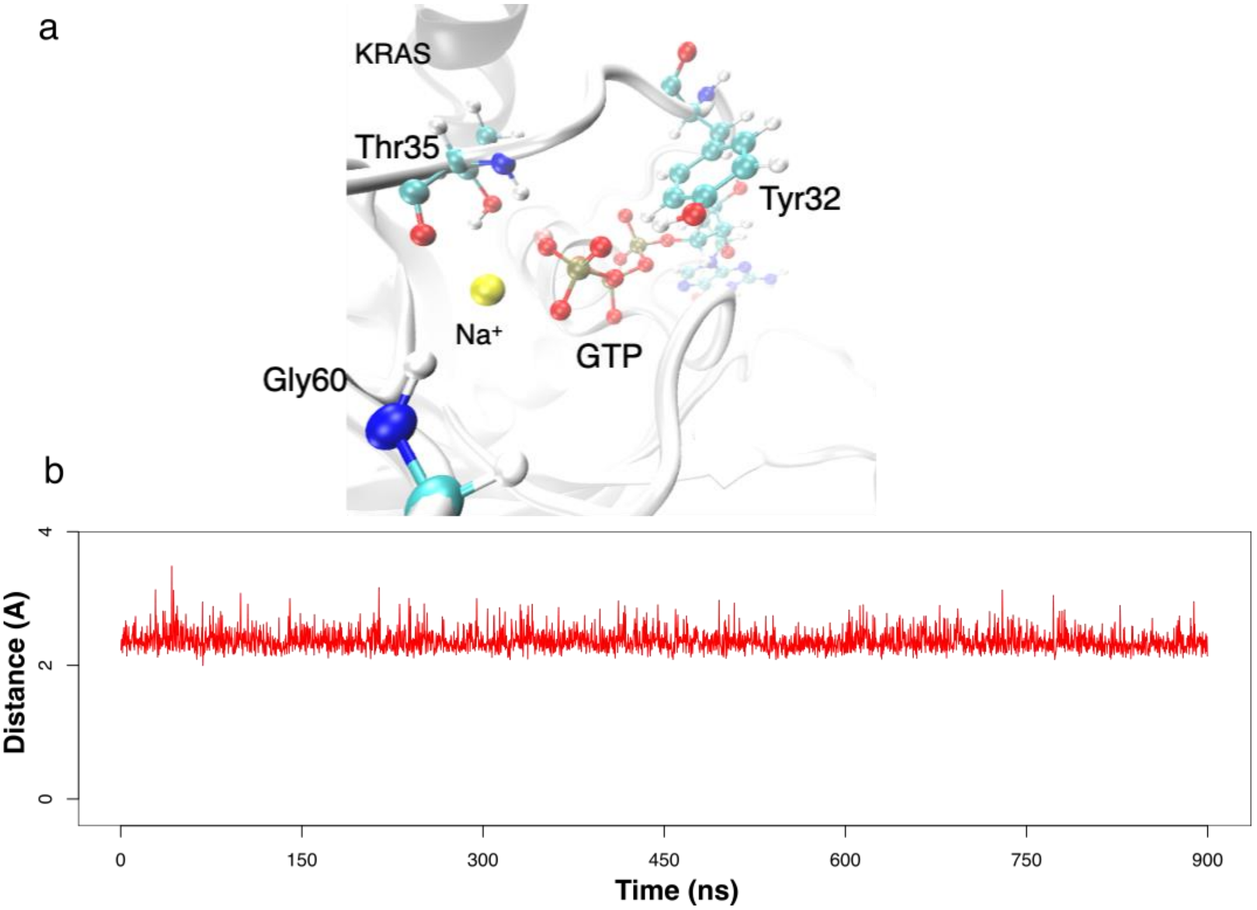

Supplementary Fig. 16: Tyr 32 of wild-type KRAS interacts with the gamma phosphate of GTP in the KRAS:RAF1 complex. **(a)** Cartoon representation of the orientation of Tyr32 and a long-lasting interaction with a Na^+ ion. **(b)** Time evolution of the distance between the long lasting Na^+ ion and the oxygen atom of the gamma phosphate.

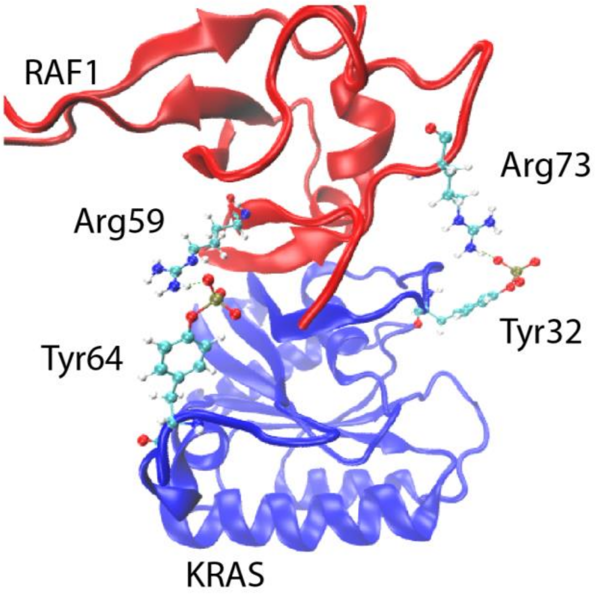

Supplementary Fig. 17: Phosphorylated Tyr32 and Tyr64 side chains form new interactions with RAF1. A snapshot of the KRAS Q61H-RAF1 complex illustrating the predicted interactions between phosphorylated Tyr32 and Tyr64 side chains and Arg73 and Arg59 of RAF1. . Selected hydrogen bonds are indicated with dotted green lines.

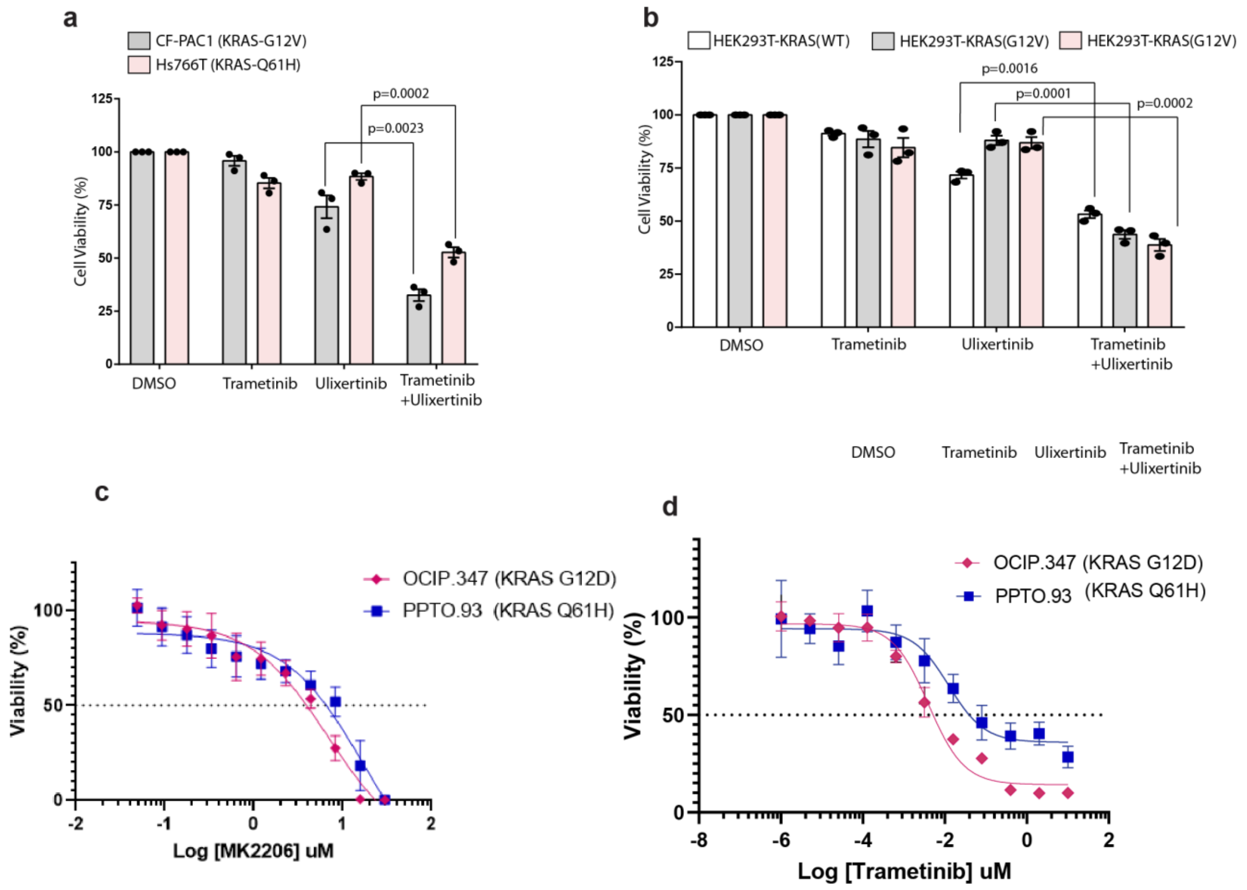

Supplementary Fig. 18: Cell viability assays of *KRAS*-mutant PDAC cells in response to inhibitors of MAPK signaling., Cell viability was assessed using alamarBlue of **(a)** CF-PAC1 and Hs766T, or **(b)** HEK-293T cells overexpressing KRAS wild-type or mutant proteins, following treatment with DMSO, trametinib [0.15 µM], ulixertinib [0.5 µM], or trametinib plus ulixertinib for 72 hours. Error bars represent s.e.m of three independent experiments. The p-values where obtained using two sided Student's t-test. **(c,d)** PDAC organoids cells were plated in 96-well plates in triplicate, treated with increasing concentrations of MK2206 **(c)** and Trametinib **(d)**for 96 h and then cell viability was determined using CellTiter-Glo 3D viability assay (Promega, Madison, USA). Individual points are shown as the mean of three (Trametinib) or six (MK2206) experiments with error bars representing s.e.m. of three independent experiments.

Supplementary Table 1: Nucleotide exchange and hydrolysis properties of KRAS and selected mutants. **(a)** Intrinsic and SOS^{cat}-assisted nucleotide exchange rates. **(b)** Intrinsic and RASA1 GAP-assisted GTP hydrolysis rates of wild-type and Q61H mutant KRAS (1:3000 KRAS:GAP ratio). The data were obtained using real-time NMR and fitted with one-phase exponential decay function.

a)

	WT	G12V	G12D	G12C	G13D	Q61L	Q61H
Intrinsic k (min^{-1}) x 10^3	2.1 ± 0.35	3.4 ± 1.8	3.5 ± 1.8	2.1 ± 0.8	26.7 ± 1.6	8.0 ± 0.8	3.2 ± 0.9
SOS^{cat} (1:600) k (min^{-1}) x10^3	42.2 ± 7.9	29.7 ± 15.9	17.6 ± 6.0	4.8 ± 0.9	49.2 ± 7.8	26.3 ± 3.2	3.1 ± 0.5

b)

WT k (min^{-1}) x 10^3		Q61H k (min^{-1}) x 10^3	
Intrinsic	RASA1 GAP	Intrinsic	RASA1 GAP
7.5 ± 1.7	14.1 ± 1.6	2.2 ± 0.6	1.3 ± 0.6

Supplementary Table 2: Impact of tyrosyl phosphorylation and Q61H mutation on KRAS nucleotide exchange and hydrolysis. **(a)** Intrinsic and SOS^{cat}-assisted nucleotide exchange rates, and **(b)** Intrinsic and RASA1 GAP-assisted GTP hydrolysis rates unmodified and phosphorylated wild-type KRAS and Q61H. The data was obtained using real-time NMR and fitted with one-phase exponential decay function.

a)

	WT	pWT	Q61H	pQ61H
Intrinsic k (min^{-1}) x 10^3	1.89 ± 0.04	7.55 ± 1.5	3.2 ± 0.1	18.2 ± 2.5
SOS^{cat} (1:600) k (min^{-1}) x10^3	46.12 ± 7.8	21.35 ± 3.7	3.1 ± 0.3	18.9 ± 1.5

b)

WT k (min^{-1}) x 10^3		pWT k (min^{-1}) x 10^3		Q61H k (min^{-1}) x 10^3		pQ61H k (min^{-1}) x 10^3	
Intrinsic	RASA1 GAP	Intrinsic	RASA1 GAP	Intrinsic	RASA1 GAP	Intrinsic	RASA1 GAP
7.5 ± 1.7	14.1 ± 1.6	1.96 0.24	2.55 ± 0.24	0.8 ± 0.08	0.8 ± 0.17	0.46 ± 0.06	0.63 ± 0.24

Supplementary Table 3: PDB accession codes of protein coordinates used as starting structures for molecular dynamics simulations.

**Molecular dynamics system**	**PDB ID of the starting structure**	**Remarks**
KRAS:SOS and KRASQ61H: SOS	6EPL(KRASG12C:SOS) [1]	The KRASG12C:SOS complex was mutated to derive models of wild-type KRAS:SOS and KRAS-Q61H:SOS
KRASQ61H:RasGAP complex	6GOG (KRAS Q61H) [2] 1WQ1 (KRAS complex with RasGAP) [3]	1WQ1 is a crystal structure of HRAS in complex with RasGAP. The KRAS-Q61H: RasGAP complex was modeled by replacing HRas with the KRASQ61H structure from PDB ID 6GOG. GDP and AlF4 were substituted with GTP
KRAS: RAF1 and KRASQ61H:RAF1 pKRAS:RAF1 pKRASQ61H:RAF1	6GOD(KRAS) [2] 6GOG (KRAS Q61H) [2] 4G0N (HRAS in complex with RAF1) [4]	HRAS from 4G0N was replaced by KRAS (GOD) or KRASQ61H (6GOG). GTP was substituted for GMPPMP in 6GOG and 6GOD

Supplementary Table 4: Hydrogen bonds exhibiting more than 20% occupancy in at least one of the complexes between KRAS and RAF1-RBD.

KRAS	RBD	WT-KRAS	pWT-KRAS	KRASQ61H	pKRASQ61H
GLU37-Side	ARG59-Side	72.91%	-	63.17%	77.47%
ASP38-Side	THR68-Side	69.54%	61.78%	68.73%	64.81%
ASP38-Side	ARG89-Side	64.98%	71.11%	64.54%	60.08%
GLU37-Side	ARG67-Side	44.79%	55.21%	49.88%	43.75%
ASP33-Side	ARG73-Side	42.92%	44.52%	66.80%	-
SER39-Main	ARG89-Side	41.29%	52.08%	40.46%	36.69%
GLU63-Side	THR54-Main	38.12%	-	-	-
SER39-Main	ARG67-Main	31.26%	28.19%	21.38%	-
GLU31-Side	LYS84-Side	30.12%	48.15%	-	43.45%
SER39-Side	GLN66-Side	30.02%	-	-	-
ASP33-Side	LYS84-Side	29.69%	20.13%	-	41.92%
TYR32-Side	ARG73-Side	-	35.89%	-	-
TYR64-Side	ARG59-Side	-	32.76%	-	-
TYR64-Side	THR54-Main	-	29.29%	-	-

-hydrogen bonds were either not observed or have occupancy less than 20 %

Supplementary Table 5: Primer sequences for quick change mutation and CRISPR/Cas9-mediated gene editing

Mutation/gene	5’ Forward 3’
KRAS G12V	GTGGTGGGCGCGGTGGGCGTGGGCAAAAG
KRAS G12C	GTGGTGGGCGCGTGTGGCGTGGGCAAAAG
KRAS G12D	GTGGTGGGCGCGGATGGCGTGGGCAAAAG
KRAS G13D	GTGGTGGGCGCGGGCGATGTGGGCAAAAG
KRAS Q61L	GATACCGCGGGCCTGGAAGAATATAGCGCG
KRAS Q61H	GATACCGCGGGCCATGAAGAATATAGCGCG
KRAS Y32F	CAGAACCATTTTGTGGATGAATTTGATCCGACCATTGAAG
KRAS Y64F	CGCGGGCCAGGAAGAATTTAGCGCGATGCGCGATC
KRAS Y40F	CATTGAAGATAGCTTTCGCAAGCAGGTGGTGATTG
Exon 1 of SHP2 (human)	TACAGTACTACAACTCAAGC
non-target control sequence	ACGGAGGCTAAGCGTCGCAA.

Supplementary References

1 Hillig, R. C. *et al.* Discovery of potent SOS1 inhibitors that block RAS activation via disruption of the RAS-SOS1 interaction. *Proc Natl Acad Sci U S A* **116**, 2551-2560, doi:10.1073/pnas.1812963116 (2019).

2 Cruz-Migoni, A. *et al.* Structure-based development of new RAS-effector inhibitors from a combination of active and inactive RAS-binding compounds. *Proc Natl Acad Sci U S A* **116**, 2545-2550, doi:10.1073/pnas.1811360116 (2019).

3 Scheffzek, K. *et al.* The Ras-RasGAP complex: structural basis for GTPase activation and its loss in oncogenic Ras mutants. *Science* **277**, 333-338, doi:10.1126/science.277.5324.333 (1997).

4 Fetics, S. K. *et al.* Allosteric effects of the oncogenic RasQ61L mutant on Raf-RBD. *Structure* **23**, 505-516, doi:10.1016/j.str.2014.12.017 (2015).
