# Supplementary material for: The Q61H mutation decouples KRAS from upstream regulation and renders cancer cells resistant to SHP2 inhibitors
Source: Nat Commun. 2021 Nov 1;12:6274. doi: 10.1038/s41467-021-26526-y (PMC8560773; doi:10.1038/s41467-021-26526-y)
Supplement: Supplementary file 3 — Reporting Summary [file 41467_2021_26526_MOESM3_ESM.pdf]

nature portfolio

Corresponding author(s): Christopher B. Marshall
Michael Ohh
Mitsuhiko Ikura

Last updated by author(s): Aug 25, 2021

Reporting Summary

Nature Portfolio wishes to improve the reproducibility of the work that we publish. This form provides structure for consistency and transparency in reporting. For further information on Nature Portfolio policies, see our Editorial Policies and the Editorial Policy Checklist.

Statistics

For all statistical analyses, confirm that the following items are present in the figure legend, table legend, main text, or Methods section.

n/a	Confirmed	
☐	☒	The exact sample size (n) for each experimental group/condition, given as a discrete number and unit of measurement
☐	☒	A statement on whether measurements were taken from distinct samples or whether the same sample was measured repeatedly
☐	☒	The statistical test(s) used AND whether they are one- or two-sided<br>*Only common tests should be described solely by name; describe more complex techniques in the Methods section.*
☒	☐	A description of all covariates tested
☐	☒	A description of any assumptions or corrections, such as tests of normality and adjustment for multiple comparisons
☐	☒	A full description of the statistical parameters including central tendency (e.g. means) or other basic estimates (e.g. regression coefficient) AND variation (e.g. standard deviation) or associated estimates of uncertainty (e.g. confidence intervals)
☐	☒	For null hypothesis testing, the test statistic (e.g. F, t, r) with confidence intervals, effect sizes, degrees of freedom and P value noted<br>*Give P values as exact values whenever suitable.*
☒	☐	For Bayesian analysis, information on the choice of priors and Markov chain Monte Carlo settings
☒	☐	For hierarchical and complex designs, identification of the appropriate level for tests and full reporting of outcomes
☒	☐	Estimates of effect sizes (e.g. Cohen's d, Pearson's r), indicating how they were calculated

Our web collection on statistics for biologists contains articles on many of the points above.

Software and code

Policy information about availability of computer code

Data collection	NMR data was collected using Bruker TopSpin 3.2.<br>BLI data was collected using Octet Data Acquisition 9.0.0.37 (ForteBio).<br>MD simulation was conducted using NMAD 2.13
Data analysis	GraphPad PRISM 6.0 software was used to analyze cell/organoid viability and tumor progression data.<br>Densitometric quantification of immunoblots was performed with imageJ software<br>NMR data were processed with NMRPipe and analyzed using NMRFAM-SPARKY, and kinetics were analyzed using GraphPad Prism 7.0.<br>BLI data was analyzed with OCtet Data Analysis 9.0.12(ForteBio).<br>Mass spectrometry of intact proteins were collected and analyzed using MassHunter Qual B.0.6.0 with Bioconfirm.<br>Phosphoproteomics data were analyzed using Proteowizard (v3.0.10800) and XITandem (v2013.06.15.1)<br>VMD 1.9.4 and Bio3d were used for MD data analysis

For manuscripts utilizing custom algorithms or software that are central to the research but not yet described in published literature, software must be made available to editors and reviewers. We strongly encourage code deposition in a community repository (e.g. GitHub). See the Nature Portfolio guidelines for submitting code & software for further information.

Data

Policy information about availability of data

All manuscripts must include a data availability statement. This statement should provide the following information, where applicable:

- Accession codes, unique identifiers, or web links for publicly available datasets
- A description of any restrictions on data availability
- For clinical datasets or third party data, please ensure that the statement adheres to our policy

Mass spectrometry data is available in the MassIVE repository (https://massive.ucsd.edu) under accession number MSV000084657.

Field-specific reporting

Please select the one below that is the best fit for your research. If you are not sure, read the appropriate sections before making your selection.

☒ Life sciences ☐ Behavioural & social sciences ☐ Ecological, evolutionary & environmental sciences

For a reference copy of the document with all sections, see nature.com/documents/nr-reporting-summary-flat.pdf

Life sciences study design

All studies must disclose on these points even when the disclosure is negative.

Sample size	For biochemical assays, at least three separate experiments were performed. Real-time NMR based nucleotide exchange and hydrolysis assays were performed at least in duplicate. All Western Blots and cell viability assays were performed in triplicate.
Data exclusions	No data were excluded
Replication	All attempts at replication were successful
Randomization	Cell lines, PDX cell, spheroids and organoids were randomized into treatment groups (control versus SHP2 inhibitor).
Blinding	Inhibitor studies were analyzed independently by two investigators

Reporting for specific materials, systems and methods

We require information from authors about some types of materials, experimental systems and methods used in many studies. Here, indicate whether each material, system or method listed is relevant to your study. If you are not sure if a list item applies to your research, read the appropriate section before selecting a response.

Materials & experimental systems

n/a	Involved in the study	
☐	☒	Antibodies
☐	☒	Eukaryotic cell lines
☒	☐	Palaeontology and archaeology
☒	☐	Animals and other organisms
☒	☐	Human research participants
☒	☐	Clinical data
☒	☐	Dual use research of concern

Methods

n/a	Involved in the study	
☒	☐	ChIP-seq
☒	☐	Flow cytometry
☒	☐	MRI-based neuroimaging

Antibodies

Antibodies used	anti-pRAS Y64 rabbit polyclonal antibody was produced for this study by BETHYL Laboratory Inc. (1:1000). Pan-Ras (OP40, 1:500), monoclonal antibodies against HA (12CA5, 1:500), KRAS (OP24, 1:200, 1:500) and pTyr (4G10) (05-321, 1:1000) were obtained from Boehringer Ingelheim and Millipore, respectively. Monoclonal FLAG-M2 (F1804, 1:2000),), beta-actin (A5316, 1:2000) and Vinculin (V9264, 1:2000) antibodies were obtained from Sigma. Rabbit polyclonal antibodies against Src (#2109, 1:5000), pERK (#9101, 1:1000), ERK (#9102, 1:1000), pTyr (P-Tyr-1000) (#8954, 1:2000), Ras (3965S, 1:1000), and HA (#3724, 1:5000) were obtained from Cell Signaling Technologies. HA.11 (#16B12, 1:1000) was obtained from Covance. Polyclonal IgG (sc-2027), HRAS (sc-520, 1:1000), NRAS (sc-519, 1:1000) and SHP2 (sc-280, 1:1000) were obtained from Santa Cruz Biotechnology.
Validation	All commercial antibodies used in this study have been validated by the manufacturers from whom they were purchased. The antibody generated against KRAS pTyr64 was validated to recognize only the phospho-Tyr64 form of KRAS by Western blotting Src-phosphorylated versus un-phosphorylated protein, mutagenesis of KRAS Tyr residues, and competition by pTyr64

Eukaryotic cell lines

Policy information about cell lines

Cell line source(s)	HEK293, CFPAC1, Capan-1, HPAF-II, SW1990, HUPT3, MiaPaCa-2, and Hs766T cells were obtained from the American Type Culture Collection. P411T1 were generated from PDAC patient-derived xenografts. OCIP.236, OCIP.347, and PPT.93 were generated by the Princess Margaret Living Biobank (PMLB) Organoid core facility (https://www.livingbiobank.ca, Toronto, Canada).
Authentication	All cell lines were verified for purity using STR analysis.
Mycoplasma contamination	All cell lines tested negative for mycoplasma contamination.
Commonly misidentified lines (See ICLAC register)	N/A
